# Speciation of Uranium and Plutonium From Nuclear Legacy Sites to the Environment: A Mini Review

**DOI:** 10.3389/fchem.2020.00630

**Published:** 2020-08-12

**Authors:** Anna Yu. Romanchuk, Irina E. Vlasova, Stepan N. Kalmykov

**Affiliations:** Department of Chemistry, Lomonosov Moscow State University, Moscow, Russia

**Keywords:** plutonium, uranium, migration, environment, speciation

## Abstract

The row of 15 chemical elements from Ac to Lr with atomic numbers from 89 to 103 are known as the actinides, which are all radioactive. Among them, uranium and plutonium are the most important as they are used in the nuclear fuel cycle and nuclear weapon production. Since the beginning of national nuclear programs and nuclear tests, many radioactively contaminated nuclear legacy sites, have been formed. This mini review covers the latest experimental, modeling, and case studies of plutonium and uranium migration in the environment, including the speciation of these elements and the chemical reactions that control their migration pathways.

## Introduction

The discovery of the nuclear fissions of U-235 and Pu-239 led to the era of nuclear weapons development and nuclear energy production. The first national nuclear programs dealt with uranium separation from ore, U-235 enrichment, plutonium production, and fuel fabrication without considering the radiation safety and environmental protection. From the start of nuclear programs and up to the period of environmental awareness, 1940s to the 1980s, many nuclear sites were established worldwide that to this day are contaminated with uranium and plutonium (Rybalchenko et al., 2005; Novikov et al., 2006; Zachara et al., 2013). Every nuclear fuel production and waste reprocessing site in the 1940–1960s used surface or underground radioactive waste storage. Many countries adopted strategies for the remediation of contaminated sites, and in some cases, remediation has been started or even completed (Conradson et al., 2011). The speciation of radionuclides in contaminated sites has been studied, and recently new discoveries were made about the speciation of uranium and plutonium that were released into the environment as a result of accidents or in the course of daily operation. Sufficient time has passed since these sites were established to clarify the migration processes. Today, we have 60 years of data on nuclear test sites and 30 years of data on consequences of the Chernobyl nuclear power plant (NPP) accident. Analysis of these data may help us to better understand the environmental behavior of actinides.

**Figure 1:**
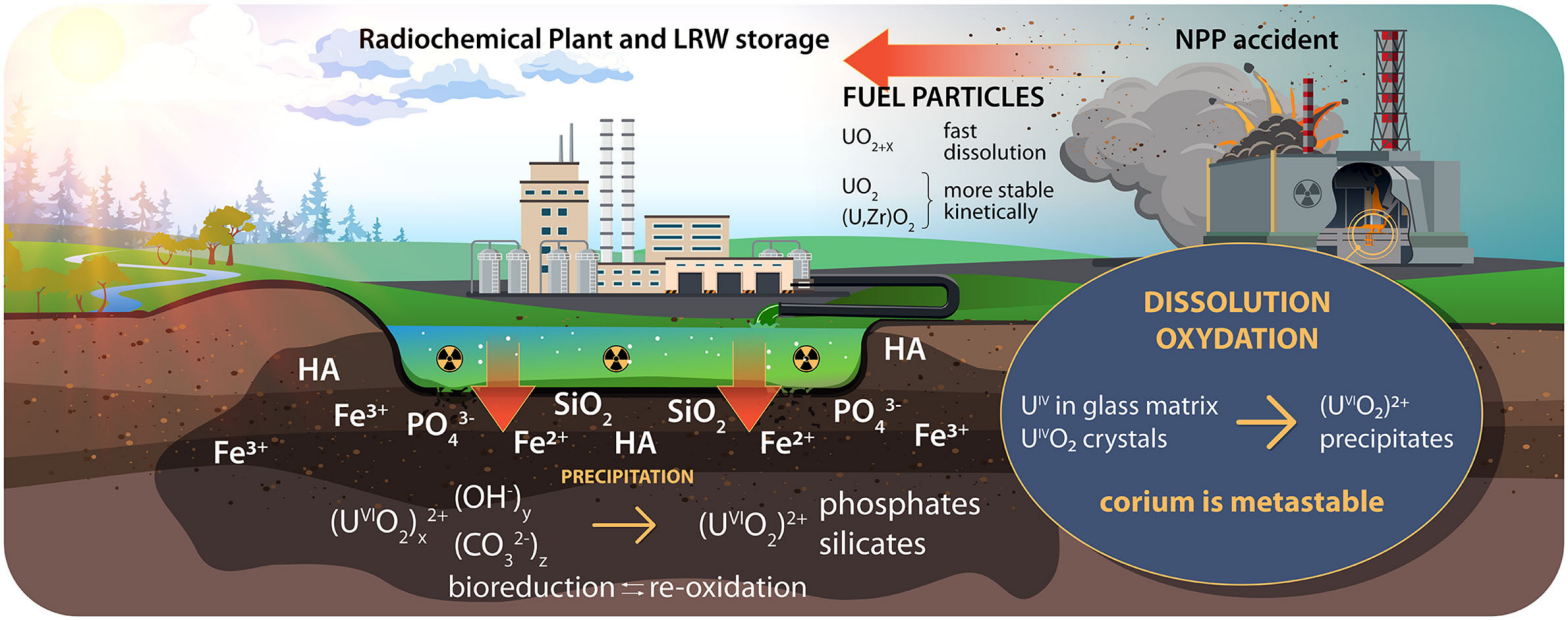
U migration into the environment. NPP, nuclear power plant; LRW, liquid radioactive waste; HA, humic acid.

**Figure 2:**
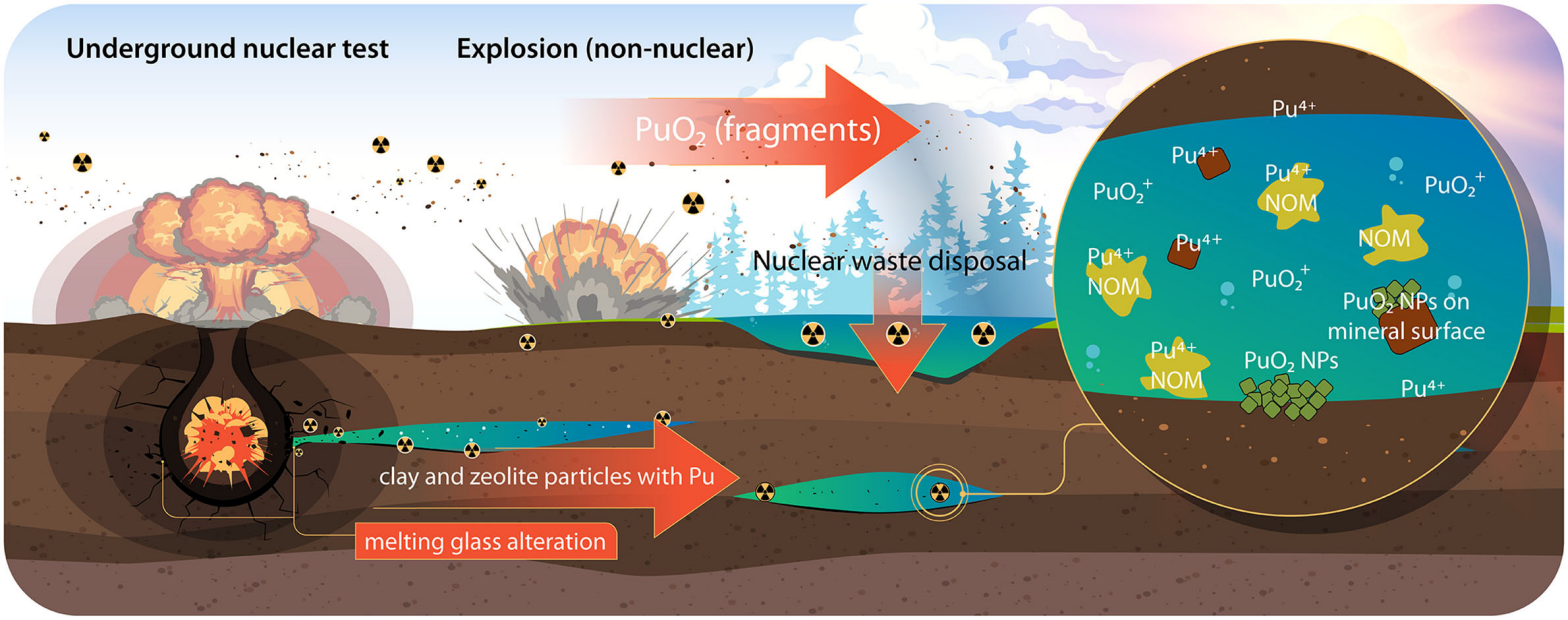
Pu migration into the environment. NP, nanoparticle; NOM, natural organic matter.

We review the recent trends in the studies of the physics and chemistry of uranium and plutonium in the nuclear legacy sites and in laboratory experiments. Figures 1, 2 summarize the speciation of U and Pu and the migration pathways of U and Pu from the legacy sites into the environment.

## Uranium

Two main oxidation states of uranium, namely +4 and +6, exist under natural geochemical conditions. In aqueous solutions, uranium is mainly present in its most mobile form the uranyl cation U^VI^${\text{O}}_{2}^{2+}$, typically in the form of hydroxyl and carbonate complexes. The mobility of uranium depends on the redox conditions and the presence of cations (Ca^2+^, Fe^2+^, and Fe^3+^), anions (carbonate, phosphate, and sulfate), silica, natural rock-forming minerals, organic substances, and microorganisms (Figure 1). In a reducing environment (oxygen-free conditions of deep underground disposals in the presence of microorganisms), uranium can transform into a less mobile form, U(IV). However, U(IV) may easily reoxidize if the conditions change. At the same time, uranium could be kinetically stabilized in “hot” particles. For example, as a result of accidental fallout (e.g., the Chernobyl accident), kinetically stable fuel particles were released into the environment, in which U(IV) was mainly in the form of UO_2_ (Figure 1).

For a long time, it was believed that pentavalent uranium does not occur in nature with a few exceptions since the cation U^V^${\text{O}}_{2}^{+}$ is unstable in aqueous solution due to its disproportionation into U^IV^ species and U^VI^${\text{O}}_{2}^{2+}$ (Arnold et al., 2009). In addition, a technical difficulty occurred in detecting pentavalent uranium in the samples. The latest U L_3_ X-ray absorption spectroscopy (XANES) study demonstrates U^VI^ reduction to U^V^ in the presence of Fe^II^/Fe^III^ oxides and oxy-hydroxides (Boland et al., 2014; Marshall et al., 2015). Three valence states of uranium, U(IV), U(V), and U(VI), could be distinguished via U M_4_ edge high-energy resolution X-ray absorption spectroscopy (HERFD-XANES) in a sample with a U concentration range of 1,000–10,000 ppm (Pidchenko et al., 2017; Roberts et al., 2017). Therefore, U(V) should also be considered in the environment.

### Immobilization of U

Uranium leakage from near-surface and surface liquid radioactive waste (LRW) storage facilities (Figure 1) as well as from underground deep disposals into permeable formations occurs mainly via the release of uranyl species. Moreover, in contaminated soils from the sites of uranium mining, milling and enrichment, uranium is also found mainly in the uranyl form. The mobility of hydroxo and carbonato uranyl complexes in aqueous systems is a severe problem for many nuclear legacy sites. The mechanisms of uranyl retention via sorption, incorporation, precipitation, and reduction, among other processes, are being studied under simulated natural and waste disposal conditions. In addition to laboratory modeling, the most effective strategies for U immobilization are identified via field tests and case studies.

The remediation of contaminated soils faces the problem of selecting a technical strategy. This is complicated by the factors that affect the behavior of uranium, which include the soil type, the mineral composition, the natural organic matter (OM), and the initial forms of uranium; see, for example Selvakumar et al. (2018) and references therein. In the case of uranium contamination of the vadose zone, especially in arid and semiarid climates, there are two main ways to prevent uranium from entering groundwater in quantities that exceed acceptable levels: by monitoring natural attenuation (e.g., through adsorption processes or radioactive decay) or by remedial actions (e.g., mass or mobility reduction of uranium) (Dresel et al., 2011). The Hanford site's natural-technological system is one of the most studied in terms of uranium behavior in the interactions of acidic, neutral, and alkaline wastes with deposits (Szecsody et al., 2013; Gartman et al., 2015). At two contaminated sites, Hanford 300A (Columbia River) and Rifle (Colorado River), subsurface uranium plumes have been examined following the surface excavation of contaminated materials (Zachara et al., 2013). The rate of the observed uranium decrease was much lower than expected at both sites. The mechanisms that control the plume persistence were considered. At the Hanford 300A site, U(VI) that is sorbed in the vadose zone enters the oxidizing aquifer during spring water excursion, and uranium release into the groundwater is controlled by kinetically limited surface complexation. At the Rifle site, U(IV) is slowly oxidized in the suboxic groundwater. The additional influx of U(VI) from the upper vadose zone is also controlled by surface complexation; however, its contribution is local, and it is not the dominant mechanism of plume spreading. Thus, field studies show a wide variety of conditions and forms of uranium in real sites; hence, laboratory studies of the dependence of the uranium behavior on each of the environmental factors are required.

The behavior of uranyl in the presence of Ca^2+^ and ${\text{CO}}_{3}^{2-}$ has been studied. At neutral pH and low concentrations (<500 μM), U(VI) sorbs on the calcite surface with the formation of inner-sphere uranyl triscarbonate complexes, whereas at high U concentrations, U(VI) forms hydroxide/carbonato precipitates (Elzinga et al., 2004). The incorporation of uranium into the calcite and vaterite structure at the Ca^2+^ sites with aging was demonstrated in the case of at least a decimolar Ca concentration (Kelly et al., 2003, 2006; Niu et al., 2019). Experimental modeling of geological disposals under hyperalkaline calcite conditions at high U concentrations [at least 4.2 μM U(VI)] demonstrated the precipitation of calcium uranate-type phases (Mace et al., 2013; Smith et al., 2015). At low U(VI) concentrations, the formation of liebigite-like Ca_2_UO_2_(CO_3_)_3_ surface complexes and binding to calcium silicate hydrates were confirmed. The difference in uranium behavior at low concentrations under neutral and high pH was observed; in the former case, the surface complexation dominates while, in the latter, uranium mainly precipitates (Smith et al., 2015). At the same time, it was demonstrated that U(VI) sorption on orthoclase and muscovite could be reduced by the presence of Ca^2+^ at a millimolar concentration at pH > 8 by up to 30% due to the formation of the neutral aqueous Ca_2_UO_2_(CO_3_)_3_ complex (Richter et al., 2016). The study of the ternary system of Ca^2+^-U(VI)–${\text{CO}}_{3}^{2-}$ remains relevant because it covers diverse processes, such as the sorption, precipitation, and incorporation of uranium in the structure of calcium carbonate, which depend on the concentration of each member of the ternary system and the environment.

A study of the behavior of uranyl in the presence of clay minerals, one of the most common components of the environment, was conducted for U mill and mine tailing material from Saskatchewan, Canada (Schindler et al., 2015). The absorption of uranyl in the alteration zones of clay minerals and intergrowth of silicates and uranyl minerals were demonstrated along with processes of the nucleation of nanocrystals of uranyl-arsenates and silicates: cuprosklodowskite, Cu[(UO_2_)_2_(SiO_3_OH)_2_](H_2_O)_6_, and metazeunerite, Cu[(UO_2_)(AsO_4_)_2_](H_2_O)_8_.

Iron oxides and oxyhydroxides appear to significantly change the behavior of uranium, and their effects continue to be studied as the new data raises new questions. Uranyl reduction to U(V) and incorporation into Fe(II/III) oxides and oxyhydroxides in cement leachates were studied under simulated geological disposal conditions at high pH (Marshall et al., 2014, 2015) and under neutral pH (Boland et al., 2014). The uranium sorption on ferrihydrite in the presence of natural organic matter (NOM) was investigated (Dublet et al., 2017), and it was shown that the effect on uranyl sorption depended on the pH and the type of NOM.

Research on uranium speciation at contaminated sites has been followed by work on the leaching, purification, and immobilization of uranium. Various approaches have been tested and revised, and recently, many laboratory studies and field tests have been conducted with the objective of evaluating the reliability of uranium retention.

There are two reliable mechanisms for the transformation of uranium from a mobile form to a solid phase in a field case: phosphate precipitation and reduction. Both are thoroughly investigated with site samples. The mobility of uranium, which is found in deposits as the relatively soluble carbonate and hydroxocomplexes, was found to be decreased by the introduction of polyphosphate solutions, thereby resulting in the precipitation of calcium uranyl phosphate (Mehta et al., 2016). Kanematsu et al. have studied uranium speciation in the solids that precipitated from synthetic acidic to neutral wastewaters in the presence and absence of dissolved silica and phosphate using U L_III_-edge EXAFS. It was revealed that rapidly precipitated autenite [Ca(UO_2_)_2_(PO_4_)_2_·10–12(H_2_O)]-type and meta-ankoleite [K_2_(UO_2_)_2_(PO_4_)_2_·6H_2_O]-type sheets transform into phosphuranylite [K_2_Ca(UO_2_)_7_(PO_4_)_4_(OH)_6_·6H_2_O]-type sheets after 30 days of reaction. This study proved that particle size, crystallinity, and composition of the precipitates control the thermodynamic and kinetic stability of uranyl solids in sediments that are impacted by acidic/neutral waste discharges (Kanematsu et al., 2014). The forms of uranium in a similar system, uranyl-silica-phosphate, were studied by Perdrial et al. In a phosphate-free system, uranium speciation was controlled initially by the precipitation of compreignacite [K_2_(UO_2_)_6_O_4_(OH)_6_·8H_2_O]- and becquerelite [Ca(UO_2_)_6_O_4_(OH)_6_·8H_2_O]-like species. The subsequent removal of uranium coincided with that of silicon and the accumulation of boltwoodite [(K, Na)(UO_2_)_2_O_4_(HSiO_4_)_2_·0.5(H_2_O)]-like species after 180 and 365 days. In the presence of ${\text{PO}}_{4}^{3-}$, meta-ankoleite [K_2_(UO_2_)_2_O_4_(PO_4_)_2_·6H_2_O]-like species exerted direct and strong control over U speciation (Perdrial et al., 2018). The dynamics of the precipitation–sorption-phase evolution in the uranyl-phosphate system at a pH of 6 in a several-week experiment was studied in the presence of goethite and mica to simulate the environment (Munasinghe et al., 2015). Chernikovite [(H_3_O)_2_(UO_2_)_2_(PO_4_)_2_·6(H_2_O)] was the first rapidly precipitated form in the experiments with U and P while partially hydrated (meta)autenite was formed only in the presence of goethite. The transition between phases was accelerated by sorption.

The bioavailability of uranium at near-surface contaminated sites is determined by the phosphate forms of uranyl, which are easily taken up by the plants (Edayilam et al., 2020). The complexity of the uranyl-phosphate system and its practical significance, highlighted in this review, support the necessity of its further study.

Emerson et al. proved the retardation of uranium on treatment with bases, such as sodium hydroxide, ammonium hydroxide, and ammonia gas, in the presence of various minerals and natural sediments from the Hanford site (Washington State, USA) (Emerson et al., 2018). The control of the pH with ammonia gas injection may be especially useful for vadose zone environments as it does not require the addition of liquids that would increase the flux toward the groundwater. The authors suggest that the likely mechanisms for the transfer of uranium from the aqueous to the solid phase are the formation of surface coatings, coprecipitation with carbonates, and adsorption.

### Uranium and Organic Matter

Luo and Xu (2016) proved the key role of thiol groups in NOM. They are responsible for the bioremediation and immobilization of uranium when it enters the environment (soil, groundwater). Bioreduction, followed by reoxidation by oxygen, can sequestrate U via the formation of stable U-coated particles, as was shown by Li et al. (2019). The dual effects of microorganisms on the behavior of uranium (reduction or rapid reoxidation), along with biosorption, bioaccumulation, and biomineralization, are reviewed in Cumberland et al. (2016). Uncertainties that are associated with the oxidation state and mobility of uranium, which is influenced by microorganisms, on both the geological and molecular scales are reviewed in this work. Available information on the uranium-OM bond and the solubility of U-OM was collected and analyzed. During the biosorption of uranium by fungal cells, the oxidation state of uranium was unchanged while the uranium speciation did change (Günther et al., 2014). Extra- and intracellular phosphate groups are mainly responsible for uranium binding.

The interaction between uranium and NOM is especially important in wetlands. There are many examples of wetlands in which this interaction governs the partitioning (Kaplan et al., 2016, 2017; Koster van Groos et al., 2016; Mikutta et al., 2016; Schumann et al., 2017; Dublet et al., 2019; Stetten et al., 2020). One of the main characteristics of these places is the significant seasonal change. During the hot season, the wetlands become dry, and the oxidative conditions occur, while during the floods or even frozen season, the reductive conditions may change the uranium speciation. Examples around the world show all of these processes: oxidation/reduction together with precipitation/dissolution and sorption/complexation. Future research in this area could identify the relationship between the seasonal changes and the various parameters, such as the temperature, Eh, pH, [${\text{PO}}_{4}^{3+}$], [NOM], microbial and plant community and uranium behavior.

### Thermodynamic Modeling and Instrumental Techniques

Both the prediction of the uranium distribution in the environment and the selection of an efficient remediation strategy require the use of thermodynamic constants for reactions that involve U. The available databases for uranium (Grenthe et al., 1992; Guillaumont et al., 2003) lack many constants. In some cases, published constants differ significantly, and sometimes the data obtained are not in accordance with the current state of knowledge. The validation of available thermodynamic data and their refinement or the acquirement of new data are of substantial importance for investigating the behavior of uranium in nature. For example, thermodynamic data for the ternary complexation of calcium uranyl tricarbonate species CaUO_2_(CO_3_)${\;}_{3}^{2-}$ and Ca_2_UO_2_(CO_3_)_3_(aq) in the temperature range of 10–70°C were obtained in Jo et al. (2019) using time-resolved laser-induced fluorescence spectroscopy (TRLFS), ion-selective electrode potentiometry, and UV/vis adsorption spectroscopy. Thermodynamic modeling of uranium species and its comparison with the experimental data that were obtained via direct non-destructive methods is necessary to investigate of uranium behavior at the contaminated sites. The use of different databases for the same water composition leads to substantial differences in modeling as was shown by Mühr-Ebert et al. (2019). To clarify this, the researchers optimized the thermodynamic data using model calculations and laboratory tests by TRLFS, mass spectrometry, and extraction experiments under various model conditions (Mühr-Ebert et al., 2019).

The recent rapid development of spectrometric, microscopic, and other analytical methods enables us to better understand the processes that occur at the contaminated sites. Non-destructive techniques, such as scanning electron microscopy (SEM) and transmission electron microscopy (TEM) with energy-dispersive X-ray analysis (EDX), are increasingly being used together with semidestructive secondary-ionization mass spectrometry (SIMS).

In addition to the classic methods, such as TRLFS, a highly sensitive method for the identification and detection of uranium species in solids and solutions is vibration spectroscopy, including infrared absorption and Raman scattering (Lu et al., 2018). The main advantage of these techniques is the possibility of *in situ* detection and the real-time monitoring of chemical reactions that involve uranium.

A combination of experiments and calculations in the identification of uranium species yields acceptable results. For example, five uranyl aqueous complexes under a fixed U conc. (10^−5^ M) over a wide pH range from 2 to 11 were identified from the TRLFS data with parallel factor analysis, UV excitation spectra (180–370 nm) and time-dependent density functional theory. The combination of experiment and theory confirmed the presence of the [UO_2_(H_2_O)_5_]^2+^ cation (aquo-complex 1:0) and the four hydroxo-complexes (1:1, 3:5, 3:7, and 1:3) (Drobot et al., 2015).

Synchrotron radiation X-ray techniques, such as X-ray fluorescence (XRF), diffraction (XRD), and absorption spectrometry (XAFS), especially with a submicron beam, provide information on the local molecular speciation (Kelly et al., 2006; Kanematsu et al., 2014; Smith et al., 2015). Recent study of the oxidation state of uranium and the structure of electronic orbitals using resonant inelastic X-ray scattering (RIXS) and HERFD-XANES measurements at the U L_III_ (Kvashnina et al., 2015), U M_IV_ (Kvashnina et al., 2013; Butorin et al., 2016) and Pu edges (Vitova et al., 2017; Kvashnina et al., 2019) give promising results for future applications. However, a significant limitation of these experimental methods is that the uranium concentrations, even at highly contaminated sites, are often below the detection limits of advanced analytical methods. Therefore, indirect destructive methods, such as sequential extraction and solvent extraction, remain the only available methods to obtain the information.

Uranium-containing phases were identified using advanced techniques in sludges from Hanford tanks (Peterson et al., 2018). The uranium concentration in radioactive waste is much higher than in the environment of the LRW storage. A limitation of this experimental approach is that in model systems, the uranium concentrations are 10^−5^-10^−7^ mol/L while the uranium concentrations at contaminated sites are below the detection limits of advanced analytical methods. Even in studies in which XANES spectra for extremely low uranium concentrations were obtained (Zhang et al., 2016), the U content is about 40 ppm, which is not always the case for the contaminated soils. Few applications of advanced techniques for the identification of U species in the environment are available (Tayal et al., 2019). This research should be continued, but destructive analyses will inevitably have to be used to identify uranium partitioning in mobile fractions as they are often the only methods for determining the forms of uranium; see Qiao et al. (2012) and Skipperud et al. (2013).

### Uranium in “Hot” Particles and Corium

In the case of accidental fuel fallout, such as the Chernobyl fallout, uranium enters the environment initially as U(IV) species, mainly as uranium dioxide, which is partially oxidized, and sometimes as mixed uranium-zirconium dioxide (UO_2+x_, (U, Zr)O_2_) (Pöml and Burakov, 2018; Shiryaev et al., 2018) (Figure 1). The solubility of these fuel particles depends more on the conditions of their formation than on the environment (Kashparov et al., 2019). The most kinetically stable are those (U,Zr)O_2_ particles that were formed at the maximum temperature during an accident. UO_2_ particles are less stable than (U,Zr)O_2_ particles. The UO_2+x_ particles are the most soluble and the least stable. In addition to uranium, fuel particles contain fission products and transuranium elements and their behavior is associated with the behavior of the particles. According to the similarity of the chemical behaviors of Sr, Cs, and Am in the Polesie soils, these radionuclides bind to fuel particles (Bondarkov et al., 2011). For more than 30 years since the Chernobyl accident, we have traced the kinetics of the dissolution/destruction of fuel particles based on a large amount of statistical data (Beresford et al., 2016; Lecomte-Pradines et al., 2020).

Uranium can enter the environment in the form of uranium dioxide particles during an accidental discharge, such as the pollution of the Yenisei River (Bolsunovsky et al., 2017), and via regular technological influxes of liquid RW into storage pools, in which liquid RW accumulates in the bottom sediments and into sludge storage pools (Batuk et al., 2015).

A special form of uranium is corium, the molten zone of an emergency reactor. The lava-like fuel-containing materials that formed in the 4th Chernobyl NPP unit have been studied for a long time, and recently, additional analytical methods have become available to study the forms of radionuclides, such as uranium (Shiryaev et al., 2016; Burakov, 2019). The obtained data on the structures and the phase compositions of lava-like materials will facilitate the prediction of the kinetics of the mechanical destruction of “lava” and the development of best strategies for handling this type of radioactive waste, which will also be useful for handling the corium of the Fukushima Daiichi emergency reactors. Numerous core melt model studies have been conducted, and sacrificial materials have been selected (Veshchunov et al., 2008; Knebel et al., 2017).

## Plutonium

Plutonium can exist in four oxidation states: Pu(III), Pu(IV), Pu(V), and Pu(VI). These states can even exist simultaneously under suitable environmental conditions. The redox potentials for switching between the oxidation states are close to 1 V for many reactions, which creates a unique scenario in which multiple oxidation states can coexist in solution (Clark et al., 2005). The oxidation state of plutonium controls its reactivity and solubility. Pu(VI) and Pu(V) are more soluble in water than Pu(IV) and Pu(III). The most important reactions for the environmental plutonium behavior are sorption, complexation, precipitation, and colloid formation, on all of which the oxidation state has the most profound influence.

Far fewer studies have been published on the behavior of plutonium than on that of uranium in the environment.

### Colloid Transport

The Pu migration in the environment was investigated at the Nevada National Security Site (NNSS), where 828 underground nuclear tests were conducted, and Pu was found in two aquifers, 1.3 km away from a nuclear test location (Kersting et al., 1999). Over 95% of the Pu in the groundwater well was associated with colloidal particles (mainly clays and zeolites). In recent years, the mechanism of the formation of these Pu species has been deeply clarified. It was found that alteration of the NNSS nuclear melt glass under hydrothermal conditions that likely occurred in the vicinity and aftermath of the underground nuclear explosion resulted in the formation of mineral colloids, which are similar to those that were reported in 1999, and plutonium was predominantly associated with them (Zavarin et al., 2019) (Figure 2). It was concluded that colloid-bound Pu was produced as a result of nuclear melt glass aging, which suggests that the local geochemistry of the test site may not be very important in the first stages of the migration. The authors attempted to describe the subsequent leaching of plutonium from these colloids. The results suggest that the hydrothermal conditions during colloid formation affect the Pu desorption rates from the colloids (Joseph et al., 2019). The desorption rates of Pu from the colloids that were formed at 140°C can be modeled based on the kinetics of Pu sorption/desorption onto montmorillonite at room temperature. The authors estimate that the half-life of Pu on colloids is on the order of 0.6–1.8 years (Begg et al., 2017). The Pu that is bound to zeolite and clay colloids at higher temperatures (≥200°C) is more stable, and the desorption rate of Pu from these colloids is at least one order of magnitude slower than that for the colloids formed by leaching at a lower temperature.

The study of the colloid transport of Pu from Lake Karachay (PA “Mayak,” Russia) (Novikov et al., 2006) found that a significant fraction of the plutonium is bound to colloids with a size <15 nm made of amorphous ferrihydrite. Many studies have been conducted on the sorption of plutonium onto mineral colloids to understand its transport. The plutonium sorption is complicated by redox reactions; hence, this topic remains open for discussion and study (Romanchuk et al., 2016b). Recently, it was found that Pu(IV) stabilization onto mineral surfaces may result in Pu surface precipitation in the form of PuO_2_ nanoparticles (Kirsch et al., 2011; Romanchuk et al., 2013, 2016a; Schmidt et al., 2013; Zhao et al., 2016) (Figure 2). Therefore, the study of PuO_2_ nanoparticles becomes increasingly important, and it is actively being conducted (Powell et al., 2011; Romanchuk et al., 2018; Kvashnina et al., 2019; Gerber et al., 2020).

The strong interaction of Pu with minerals and sediments results in stable association with no leaching is observed even after 32 years of aging (Emerson et al., 2019). In this study, solvent extraction of Pu that was bound with the surface for 3 days and for 32 years shows that the aging changes the speciation of Pu on the surface; however, the exact mechanism of the aging remains undefined. Understanding of the aging and of the stronger Pu binding with minerals or colloids is important for the prediction of Pu transport in the environment.

### Plutonium and NOM

Most of the plutonium that was released into the environment during the Chernobyl accident was associated with uranium particles. The subsequent migration of this plutonium was controlled by the dissolution of these particles; see above. The plutonium concentration in the groundwater of the Chernobyl Exclusion Zone remains low (Bugai et al., 2020). However, these concentrations are much higher than those that are determined via simple calculations that apply the distribution coefficient (*K*_d_) concept. A relatively low *K*_d_ of plutonium was obtained for the *in situ* experiments compared to the values that were reported for a batch. The mechanism of this overstated plutonium migration remains to be identified. It can most likely be explained by facilitated transport in the form of mobile colloids or of complexes with NOM, especially considering the local geochemistry. For example, (Levchuk et al., 2012) demonstrated using ultrafiltration that a large fraction of the plutonium in the groundwater of the “Red forest” (5 km from the Chernobyl NPP) is bound with low-molecular-weight NOM (<1 kDa) and humic and fulvic acids.

The influence of organic substances on plutonium migration has been discussed in other works (Zhao et al., 2011; Santschi et al., 2017; Lin et al., 2019). In the NNSS vadose zone, the water that was collected in a tunnel system had plutonium predominantly as an aqueous species, which was not bound with colloids (Zhao et al., 2011). It was found that more than 70% of the plutonium was complexed with dissolved organic matter and that its high content (15–19 mg/L) could be attributed to anthropogenic mining activity.

Another example of the influence of organic matter on the behavior of Pu was found in the Nishiyama reservoir (Nagasaki, Japan) (Lin et al., 2019). Nagasaki is the only city in the world where a Pu bomb was exploded. As a result, an area of 3–5 km^2^ in Nagasaki was highly contaminated by 200 radioactive isotopes. Plutonium precipitated to the earth as a “black rain,” which is a mixture of soot carbon and water. High levels of plutonium remain in the sediments of the Nishiyama reservoir. Most of the Pu in the sediments was preferentially bound to the organic matter that was stabilized by Fe oxides. The authors conclude that the chelation of Pu by the hydroxamate siderophores occurs in the sub-/surface soils, similar to the F-area of the Savannah River site and the Fukushima prefecture (Santschi et al., 2017). Santschi et al. proposed that Pu(IV) may become even more mobile than oxidized Pu(V/VI). As a result, Pu(IV) bound with organic matter can form mobile colloids, especially at higher pH. Therefore, NOM can immobilize or remobilize Pu depending on the pH. This effect should be considered in the migration modeling and should be studied further. The data on the influence of NOM on the interaction of Pu with minerals and sediments are limited.

### Pu in “Hot” Particles

Synchrotron-based techniques, especially with microresolution, may help in the speciation of Pu-containing particles. A valuable example is the remediation of the Rocky Flats environmental technology site. A combination of XANES and EXAFS methods facilitated the detection of PuO_2_-like particles in the soil (Conradson et al., 2011). These particles exhibit slow leaching with no production of secondary forms of plutonium. Thus, the remediation process could be made cheaper and easier.

Another example is a study of Pu-containing particles at the Taranaki site, which is in southern Australia, where 500 non-nuclear explosions were conducted in 1953–1963 (Ikeda-Ohno et al., 2016). Applying modern μ-XRF and μ-XANES techniques, the authors determined that the initial detonation of the weapon components resulted in the formation of PuO_2_ particles that contain Pb. However, storage in the soil and the environmental reactions result in the precipitation or sorption on the surface of Pu particles. A core-shell structure with a Ca/Fe/U shell and a PuO_2_ core were identified by the authors. These particles can be compared with the particles formed in the accidents in Palomeras (Spain) and Thule (Greenland). In each case, the accident occurred with a B-52 bomber. In Palomeras, a non-nuclear explosion of two weapons with subsequent fire resulted in the dispersion of Pu–U particles over a 2.30 km^2^ area. In the Thule accident, a bomber crashed on the ice in the North Star Bay, 12 km west of Thule Air Base, due to a flight mission accident. This non-nuclear explosion also resulted in the dispersion of Pu–U particles in marine and terrestrial environments. The composition and properties of the particles in both accidents are similar (Lind et al., 2007). U and Pu oxide mixtures constitute the cores of the particles. The authors found that the oxidation state of U is predominately U(IV) with minor contributions from higher oxidation states (up to U_3_O_8_). Characterization of Pu shows it to be Pu(III)/Pu(IV), Pu(IV)/Pu(V), or a mixture of all three oxidation states. The particles in both accidents appear to be similar; however, their storage conditions differ. In the Thule accident, most of the particles were aged in a marine environment while a small fraction was aged in a terrestrial ecosystem that remains frozen for most of the year. In the Palomares accident, a terrestrial semi-desert ecosystem was mainly affected (Sancho and García-Tenorio, 2019). According to the leaching experiments (by 0.16 M HCl) of the EU-COMET project, the “hot” particles from the Thule sediment are more leachable than those from the Palomares soils (Vandenhove et al., 2017). These data demonstrate that the weathering of “hot” particles and their evolution over time may depend not only on the contamination scenario, but also on the storage conditions.

### Pu in Waste Storage

A more complicated scenario is found in the radioactive waste disposal sites and in the pathways of migration from them. Many studies have been conducted on the temporary storage or disposal of liquid and solid radioactive wastes. The composition of the radioactive waste and the environmental conditions of disposal differ substantially from one disposal to another. Unfortunately, only a few of them are open for scientific discussion.

Recently, the migration of radionuclides from a legacy trench disposal site in eastern Australia was investigated (Payne et al., 2020). The authors studied the migration of actinides and fission products. It was found that mobilization of Pu from the trenches is related to complex interactions between the influx of oxidizing groundwater during rainfall and the subsequent extended period of reducing conditions. The microbial activity influences the distributions of actinides and other redox-sensitive elements, such as iron and manganese (Vázquez-Campos et al., 2017). The study shows seasonal changes in redox conditions, which can significantly change the migration of redox-sensitive actinides, especially Pu. The differences in seasonal changes in properties, such as the temperature and rainfall throughout the world may significantly affect the redox-sensitive migration of radionuclides.

The microbiological community, which plays a special role in these biogeochemical processes, should also be considered. The influence of the microbiological activity of radionuclide migration in groundwater was demonstrated (Kersting, 2013; Kato et al., 2020). The authors agree that the interaction of Pu with microorganisms results in a reduction, but other processes may also be involved, such as sorption onto cell surface, accumulation within the cells, biomineralization and complexation. The exact mechanism of the interaction and its influence on the Pu mobility is not clear. The interaction of Pu(V) and Pu(IV) with cells in the presence and absence of extracellular polymeric substances (EPS) was investigated (Boggs et al., 2016). It was found that the reduction of Pu(V) mostly occur in the interaction with EPS. In all studied systems, by the end of the reaction, the cells immobilize Pu on their surface. However, in other works, the reduction of Pu(IV) to Pu(III) on reaction with cells increased the mobility of the radionuclides (Xie et al., 2018).

## Outlook

The behavior of plutonium and uranium in the environment strongly depends on their source and the local geochemical conditions. In this mini review, we highlighted recent studies of the migration pathways of these radionuclides.

The research could be continued in two ways. First, the experiments under controlled laboratory conditions, which are supported by instrumental methods and calculations, may facilitate the identification of the mechanism of the reaction on the molecular level. This information is highly necessary. Thermodynamic modeling should accompany these experiments because reliable prediction of behavior of radionuclides in the environment remains impeded. In addition, long-term laboratory experiments should be conducted because aging often changes the pathways of these reactions.

Second is the characterization of environmental and the on-site experiments. Modern advanced techniques enable us to understand the chemistry of the migration at the molecular level even at the contaminated sites. However, in most cases, these methods are limited by the sensitivity. The differences between the contaminated sites means that more research should be conducted. This may facilitate the development of an economical, environmentally friendly, and sustainable solution of the remediation process.

## Author Contributions

AR and IV designed and wrote the review with input from SK regarding the conception and editing of the manuscript. All authors contributed to the article and approved the submitted version.

## Conflict of Interest

The authors declare that the research was conducted in the absence of any commercial or financial relationships that could be construed as a potential conflict of interest.
